# The Generation of Two Induced Pluripotent Cell Lines from Patients with an Atypical Familial Form of Lung Fibrosis

**DOI:** 10.3390/cells14110781

**Published:** 2025-05-26

**Authors:** Eid Al-Mutairy, Somaya M. Al Qattan, Faiqa Imtiaz, Azizah AlAnazi, Angela Inglis, Rana Al-Rabiah, Reem S. Al-Hejailan

**Affiliations:** 1 Lung Health Center, King Faisal Specialist Hospital and Research Center, Riyadh 11211, Saudi Arabia; ealmutairy45@kfshrc.edu.sa (E.A.-M.); salqattan@kfshrc.edu.sa (S.M.A.Q.); 2 Clinical Genomic Center, King Faisal Specialist Hospital and Research Center, Riyadh 11211, Saudi Arabia; fahmad@kfshrc.edu.sa; 3 Cell Biology Department, King Faisal Specialist Hospital and Research Center, Riyadh 11564, Saudi Arabiaainglis@kfshrc.edu.sa (A.I.); ranaa@kfshrc.edu.sa (R.A.-R.)

**Keywords:** atypical lung fibrosis, reprogramming, iPSCs, S100A3/S100A13, mitochondrial mutation, intracellular calcium homeostasis

## Abstract

***Background***: Pulmonary fibrosis is a major disease that leads to the progressive loss of lung function. The disease manifests early, resulting in type 2 respiratory failure. This is likely due to the bronchocentric fibrosis around the major airways, which causes airflow limitation. It affects approximately three million patients worldwide and has a poor prognosis. Skin fibroblasts isolated from patients offer valuable insights into understanding the disease mechanisms, identifying the genetic causes, and developing personalized therapies. However, the use of skin fibroblasts to study a disease that exclusively impacts the lungs is often questioned, particularly since lung fibrosis primarily affects the alveolar epithelium. ***Method***: We report the reprogramming of skin fibroblasts from patients with an atypical early-onset form of lung fibrosis into induced pluripotent stem cells (iPSCs) and subsequently into alveolar epithelial cells. This was achieved using a Sendai virus approach. ***Results***: We show that the reprogrammed cells carry mutations in the calcium-binding protein genes S100A3 and S100A13, leading to diminished protein expression, thus mimicking the patients’ cells. Additionally, we demonstrate that the generated patient iPSCs exhibit aberrant calcium and mitochondrial functions. ***Conclusions***: Due to the lack of a suitable animal model that accurately resembles the human disease, generating patient lung cells from these iPSCs can provide a valuable “disease in a dish” model for studying the atypical form of inherited lung fibrosis. This condition is associated with mutations in the calcium-binding protein genes S100A3 *(NM_002960)* and S100A13 *(NM_001024210),* aiding in the understanding of its pathogenesis.

## 1. Introduction

The fast progression of induced pluripotent stem cell (iPSC) technology has seen the transformation of in vitro research and therapeutic development [1,2,3,4,5,6,7]. Therefore, iPSC-derived cells represent an optimal model for imitating human development and disease, executing high-throughput drug screening, and assessing autologous and allogenic cell therapies. The most common iPSC-derived cell application is disease modeling [1,8,9,10,11,12,13], as iPSCs can be derived from the somatic cells of patients with disease-carrying mutations or genetic backgrounds disposing them to disease. Such iPSCs are subsequently differentiated into the affected cell types, thus emulating the disease-specific phenotypes.

“Interstitial lung diseases” (ILDs) is a broad term for a number of lung diseases that cause permanent tissue scarring or fibrosis. ILDs induce airspace structural remodeling and tissue injury along with aberrant wound healing, resulting in collagenous fibrosis and impaired gas exchange [14,15,16,17,18,19,20]. Idiopathic pulmonary fibrosis (IPF) is a progressive form of ILD that leads to respiratory failure. For these patients, a lung transplant is the only viable treatment option [21]. A number of risk factors have been found to contribute to the pathogenesis of IPF, including genetic predisposition; for example, mutations in the surfactant protein C (SFTPC) gene have implications for the familial forms of interstitial lung disease [22,23,24,25,26,27]. Familial pulmonary fibrosis (FPF) is a type of pulmonary fibrosis found in two or more first-degree relatives. It is caused by a monogenic mutation in surfactant protein A2 (AFTPA2), surfactant protein C (SFTPC), and ATP binding cassette A3 (ABCA3) [28]. Also, it was demonstrated that a gene promoter variant (rs 35705950) encoding mucin 5B (MUC5B) increases its expression and increases the risk of pulmonary fibrosis [27,29,30,31,32,33].

In this disease, the excessive accumulation of scar tissue (fibrosis) leads to a progressive decline in lung function. The accumulation of scar tissue makes it difficult for oxygen to pass through the walls of the air sacs and into the bloodstream. Type 1 pneumocytes, also known as type I alveolar cells, are one of the two main types of cell that make up the alveoli in the lungs. These cells are responsible for gas exchange, allowing oxygen to diffuse from the air into the bloodstream and carbon dioxide to diffuse out of the bloodstream into the air. While type 1 pneumocytes are not responsible for producing excess scar tissue, they can be affected by the disease and may play a role in the pathogenesis of pulmonary fibrosis. The current evidence indicates that the fibrotic response involves abnormally activated alveolar epithelial cells (AECs), beginning with damage to type I AECs (AECI), which cover the majority of the alveolar surface. When AECI are damaged, type II AECs (AECII) proliferate excessively to cover the exposed basement membrane. This process includes disordered apoptosis and the differentiation of AECII into AECI, resulting in irregular replacement and repair of the alveolar surface [34]. Additionally, the number of type 1 pneumocytes is reduced, and the remaining cells may become stretched and flattened, impairing their ability to carry out gas exchange [34]. Type I pneumocytes may also play a role in the inflammatory response in pulmonary fibrosis by secreting cytokines and other signaling molecules that attract and activate immune cells in the lung tissue [35,36]. Understanding their role in pulmonary fibrosis is crucial for developing effective treatments. Targeting these cells and their signaling pathways could provide new therapeutic strategies to slow or reverse the progression of the disease.

S100A3 and S100A13 are cation-binding proteins and are members of more than 20 different acidic, low-molecular-weight calcium- and zinc-binding proteins that exist as homodimers, heterodimers, and multioligomers [37]. The proteins display cell- and tissue-specific expression patterns, with significant structural similarities to calmodulins. Also, results have shown that patient-derived cells and human bronchial epithelial cells transfected with mutant S100A3 and/or S100A13 constitutively secrete the cytokines IL-6, IL-8, and MCP-1 [38]. In normal lung tissue, S100A3 and S100A13 expression is distributed along the ciliary edges and the apical surfaces of the bronchiolar epithelium in the small airways. However, in contrast to control lung tissue and the lung tissue from patients with sporadic IPF, the lung tissue from affected people has significantly reduced S100A3 and S100A13 expression. Recently, two variants in the calcium-binding protein genes S100A3 (*NM_002960*) and S100A13 (*NM_001024210*) were identified, segregating with the disease in seven siblings from two unrelated families with pulmonary fibrosis [39]. These mutations resulted in lower protein expression, aberrant receptor-mediated intracellular calcium responses, reduced tolerance to external oxidative stress, and altered extracellular matrix (ECM) protein expression in patient-derived cells [39,40]. Following all of the above, our group investigated a calcium-based therapy including treatment with recombinant S100A3 and S100A13 proteins. The results indicate normalized calcium changes in the patients’ cells when compared to the control cells [38,41,42,43,44,45]. Moreover, both mutants were reversed by transfecting patient-derived cells with wild-type S100A3 and S100A13, which led to an increased secretion of inflammatory mediators [40]. This reveals a potential therapeutic strategy. While the role of these proteins in fibrosis and therapy warrants further investigation, the rapid disease progression complicates patient sample studies. Therefore, a standardized disease platform is essential.

Disease modeling provides a unique opportunity to investigate the molecular and cellular mechanisms of the disease [46]. Additionally, it allows for personalized medicine by using cells from specific patients to tailor treatments to individual needs. Patient-derived cells are often used to provide a “disease in a dish” approach to the study of disease progression and response to drugs and therapies. It offers a more physiologically relevant and scalable approach compared to traditional animal models. Here, we generated induced pluripotent stem cell (iPSC) lines from two patients with pulmonary fibrosis (PF). Patients from two unrelated families, born to healthy consanguineous parents, presented with interstitial lung disease. The clinical investigations revealed the following:

Patient 1: A 27-year-old female who developed pulmonary fibrosis at age 13 and experienced dyspnea in her early teens, with no abnormalities in other medical examinations. Patient 2: A 32-year-old female who developed respiratory issues at age 13, despite having a normal appearance, development, and laboratory findings. Some of her siblings had previously died due to lung disease, and another sibling underwent a lung transplant at age 27. This sibling is currently 35 years old and in good health.

The iPSCs, when differentiated into alveolar cells, exhibited the same cellular dysfunctions as patient-derived skin fibroblasts. This approach enabled the study of mutations affecting lung cells that are directly involved in the pathogenesis of lung fibrosis.

## 2. Materials and Methods

### 2.1. Reprogramming

Idiopathic pulmonary fibrosis-Patient 1-dermal fibroblast (IPF-PT1-HDF) and Idiopathic pulmonary fibrosis-Patient 2-dermal fibroblast (IPF-PT2-HDF) were reprogrammed with the CytoTune iPSC 2.0 Reprogramming Kit (Thermofisher, A13780-1, Waltham, MA, USA) at passage two and passage three, respectively. Briefly, the HDFs were seeded in an iPSC cultivation medium [mTEsR (StemCell Tech, Vancouver, BC, Canada), 1% penicillin/streptomycin] at a density of 1 × 10^5^ cells/cm^2^ on hESC-qualified Matrigel (Corning 356231; Glendale, AZ, USA) and transfected with *KOS* (A16517/Titer 1.1 × 10^8^), *Myc* (A16517/Titer 9.9 × 10^7^), and *KIF4* (A16517/Titer1.1 × 10^8^) vectors. The cells were cultivated for 14 days, and colonies were screened for pluripotency with pluripotency live staining. Suitable colony candidates were picked and expanded further. In parallel, the FAM-iPSCs line was generated from control male skin fibroblasts following the same reprogramming and characterization protocols. The cell culture supernatant of all cultures was routinely tested for the presence of mycoplasma using a Mycoplasma PCR Detection Kit (abcam Cat# G238, Cambridge, CB4 0WH, UK). The supernatant was collected from the cells at 80% confluence 72 h prior to the test day and mixed with BlasTaq 2X PCR Mastermix; G895-ABM, Heidelberg, Germany) primers, and nuclease-free water to a volume of 25 µL. All of the samples were run on a thermocycler with positive and negative controls, as stated in the instruction manual. PCR products were run on 2% agarose gels containing ethidium bromide and visualized with a Bio-Rad GelDoc (Image Lab Software for PC Version 6.1) to detect mycoplasma. To demonstrate that the iPSCs retained S100A3 and S100A13, mutation gene sequencing was conducted on the patients’ generated iPSCs as previously described [39], utilizing Sanger sequencing. PCR was carried out with primers designed to amplify the regions containing both the S100A3 and the S100A13 variants. Sequence analysis was manually performed using the SeqMan 6.1 module of Lasergene (DNASTAR, Madison, WI, USA) with 99.05% sensitivity.

### 2.2. Pluripotency Detection

The iPSCs displayed prominent nuclei and minimal cytoplasm. Morphological assessment of the iPSC clones characterized their colonies as densely packed and flat. To investigate pluripotency, the analysis began at passage 15, involving specific staining of the iPSC clones, which confirmed the expression of the alkaline phosphatase enzyme. Three-germ-layer differentiation is a major indication of pluripotency; both generated lines, PT1- iPSCs and PT2-iPSCs, were differentiated into cell types from the endoderm, ectoderm, and mesoderm, and subsequently analyzed using immunofluorescence. Additionally, immunofluorescence staining using pluripotency markers was carried out; the cells were fixed for 20 min, washed with 1X phosphate-buffered saline solution (PBS), and then blocked with 5% fetal bovine serum (FBS) and 0.1% Triton-X for intracellular targets. Immunofluorescence staining was performed on three iPSC cell lines; primary antibodies to SOX2 (27485), SSEA4 (4755S) (Cell signaling, Danvers, MA, USA), AFP (Sigma, Darmstadt, Germany, SAB3300009), SOX17 (R&D; Minneapolis, MN, USA, AF1924), CC10 (SC-365992), EP-CAM (Sc-58806) (Santa Cruz, CA, USA; Dallas, TX, USA), NKx2.1 (ab72876), podaplanin (ab109059), α SMA (Ab7817), Nestin (ab6320), S100A3 (ab62277), and S100A13 (ab109252) (Abcam; Cambridge, UK), as well as fluorescein-conjugate secondary anti-mouse IgG and anti-rabbit IgG (ThermoFisher Scientific, Waltham, MA, USA), were used according to the manufacturers’ instructions. The nuclei were counterstained with 700 nM DAPI (Sigma Aldrich; Darmstadt, Germany, 28718-90-3). Images were acquired and analyzed using cellSens Dimension version 1.9 (Olympus, Dusseldorf, Germany).

### 2.3. Mitochondrial Integrity Measurements and Intracellular Calcium

Patients with digenic mutations in S100A3 and S100A13 are characterized by disrupted intracellular calcium homeostasis and mitochondrial dysfunction [39]. These abnormalities were detected through measurements of intracellular calcium changes in response to bradykinin (50 µM), highlighting the pivotal role of S100A3/S100A13 in receptor-induced calcium transients. Mitochondrial calcium uptake is central to these transients, playing a crucial role in intracellular calcium signaling by shaping and buffering calcium transients. Therefore, both mitochondrial integrity and intracellular calcium were measured. In a flow cytometry experiment, cells (1 × 10^6^ cells·mL^−1^) were labeled with 1 µM MitoTracker Green FM for 45 min on ice, washed (PBS, pH 7.2), fixed in 1% paraformaldehyde, and analyzed using a FACS Calibur flow cytometer (BD Biosciences) (N = 3) [39,40]. Cytosolic calcium measurements were performed on the patient ((1 × 10^6^ cell mL^−1^) or control iPSCs (1 × 10^6^ cell mL^−1^), as previously described [39,40]. Bradykinin (Sigma; 50 µM)-induced receptor-mediated changes in intracellular fluorescence intensity and ionomycin (Sigma; 2 µM) were measured using the LSM 510 META laser scanning confocal system (Carl Zeiss MicroImaging, Jena, Germany). Mitochondrial staining was performed using MitoTracker Red CMXRos 1 μM for 5 min at 37 °C (Invitrogen, Carlsbad, CA, USA) and viewed under the Yokogawa Spinning Disk confocal microscopy system (Carl Zeiss Micro Imaging (N = 6 and 5 for the control and patients’ cells, respectively).

### 2.4. Measurement of Inflammatory Mediators

The role of inflammatory mediators in the pathogenesis of lung fibrosis has previously been observed [47,48,49]. Inflammatory mediators were measured using Milliplex Map (xMAP^®^ Technology, Luminex, TX, USA) following the manufacturer’s instructions. The MILLIPLEX kits were purchased from (Millipore; Darmstadt, Germany, HCYTOMAG-60K-41, Lot # 3158918). The measurements were performed in triplicate, as described [39,40].

### 2.5. Direct Differentiation into Lung Epithelial Cells

Briefly, the patients’ iPSC cells, which had been maintained on mTESR1 media, were differentiated into definitive endoderm using the STEM diff Definitive Endoderm Kit (Cat# 05110 StemCell Technologies, Vancouver, BC, Canada), with the addition of supplements A and B for one day and supplement B only for two days. At day four, the cells were dissociated, passaged, and plated at a ratio of 1:6 in a 6-well plate coated with Matrigel in anteriorization media “DS/SB” (DMEM/F12), with 2% B27 supplement, insulin minus (Invitrogen, Waltham, MA, USA), 1% N2 supplement (Invitrogen, Waltham, MA, USA), 0.1% bovine serum, Glutamax (ThermoFisher; Waltham, MA, USA, Cat# 35050087), 10 μM SB431542 (Tocris Bioscience; Riyadh, Saudi Arabia, Catalog #: 1614), and 2 μM Dorsomorphin (Tocris; Riyadh, Saudi Arabia Cat# 3139) for an additional four days. The cells were then cultured in ventrilization media to induce a progenitor containing 3 μM CHIR99021 (Tocris; Riyadh, Saudi Arabia Cat# 4423/10), 10 ng/mL recombinant human BMP4 (R&D Systems; Minneapolis, MN, USA Cat #314-BP), and 100 nM retinoic acid (Sigma; Darmstadt, Germany Cat# R2625).

### 2.6. Statistical Analysis

ANOVA with Dunnett’s multiple comparison test was employed to assess statistical significance using Prism (GraphPad, La Jolla, CA, USA). When appropriate, an unpaired two-tailed *t*-test was utilized. A *p*-value of ≤0.05 was deemed significant.

## 3. Results

### 3.1. Generated iPSC Cell Lines

**Figure 1:**
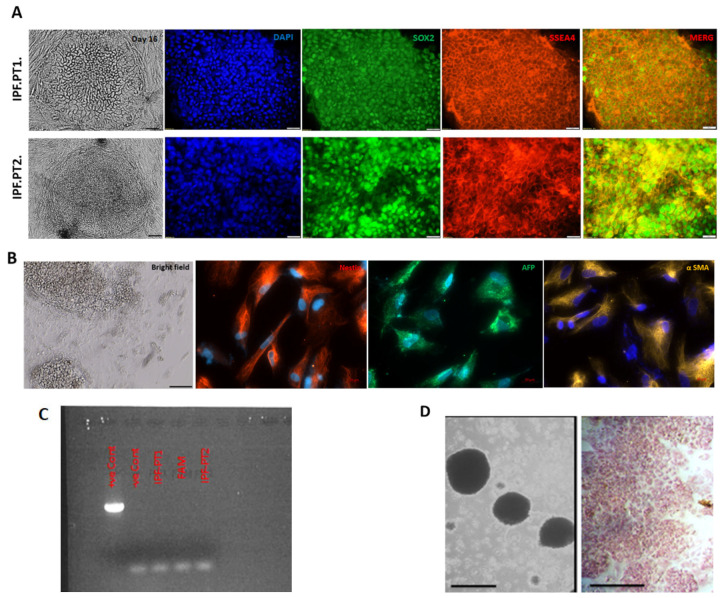
Characterization of IPF.PT1-iPSC and IPF.PT2-iPSC. (**A**) The iPSCs show normal hESC-like morphology with the expression of SOX2 and SSEA4 pluripotency markers. The nuclei were counterstained with DAPI. (**B**) The expression of endodermal alpha-fetoprotein (AFP), ectodermal Nestin, and mesodermal α-SMA, counterstained with DAPI. (**C**) Agarose gel of sample results after mycoplasma testing. (**D**) Embryoid body formation and alkaline phosphatase test at passage 15. Scale bar 10 μm.

The putative iPSCs showed a typical human embryonic stem cell-like (hESC) morphology, with clear expression of the key pluripotency markers SOX2 and SSEA4 (Figure 1A). Both iPSC lines exhibited robust expression of pluripotency markers, could be differentiated into all three germ layers (Figure 1B), and showed a positive alkaline phosphatase test. (Figure 1D). The generated cells were negative according to the mycoplasma test (Figure 1C). Molecular analyses of the IPF-PT1 and IPF-PT2 lines indicated a transition of G to A at position c.8, and arginine residue changed to Lysine at position 3 of the S100A3 protein (c.8G > A (p. Arg3Lysc)). The control cells, on the other hand, demonstrated a change of c.238–241delATTG (p. Ile80GlyfsTer13) in S100A13, as in our previous work [39,40]. This mutation is based on full-length S100A3 (NM_002960.2) and S100A13 (NM_001024211.1) transcripts, and is reflected at the protein level as well (Supplementary S1) and (Supplementary S2) [50,51,52,53,54].

### 3.2. Effects of S100A3 and S100A13 Mutations on Mitochondria and Intracellular Calcium Release

**Figure 2:**
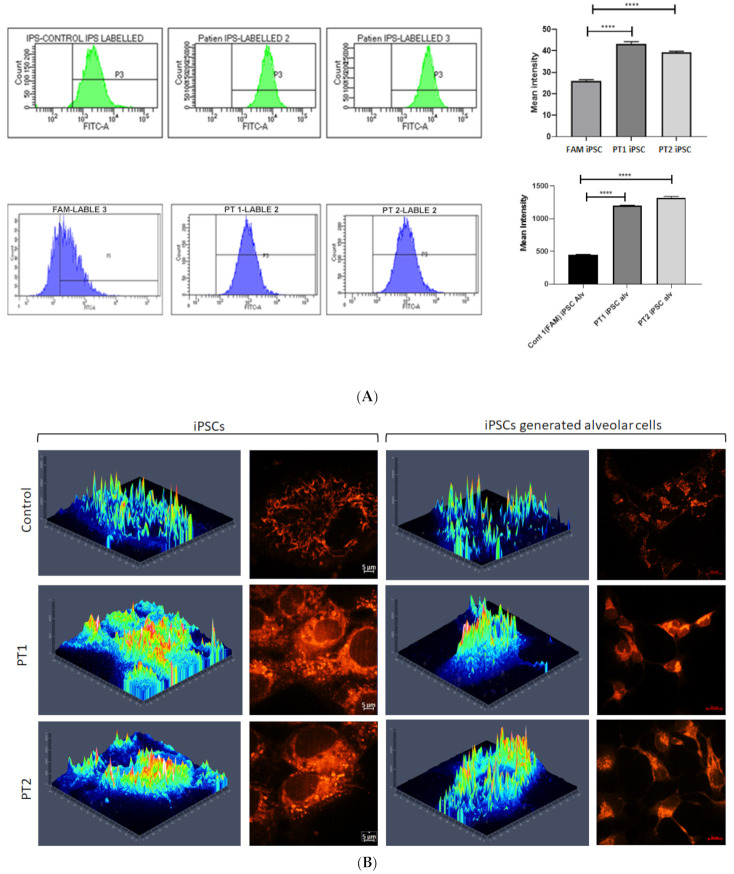
The effect of S100A3 and S100A13 mutations on mitochondria. FITC: fluorescein isothiocyanate. (**A**) Flow cytometry of the induced pluripotent stem cell- (**top**) and induced pluripotent stem cell-generated alveolar progenitors (**bottom**) from patient and control cells stained with MitoTracker Green FM. The inset shows the mean ± SEM of the fluorescence intensity in the patient and control cells. The experiments were performed in triplicate and are representative of at least three independent experiments using one million cells per sample. (**B**) Confocal fluorescence micrographs of induced pluripotent stem cell- and induced pluripotent stem cell-generated alveolar progenitors labeled with MitoTracker Red CMXRos (1 µM); the corresponding three-dimensional intensity maps are color coded so that warm colors indicate high intensity and cold colors indicate low intensity. Scale bar: 20 µm. FAM-iPSCs is a control cell line generated from control male donor skin fibroblasts. **** *p* < 0.0001.

**Figure 3:**
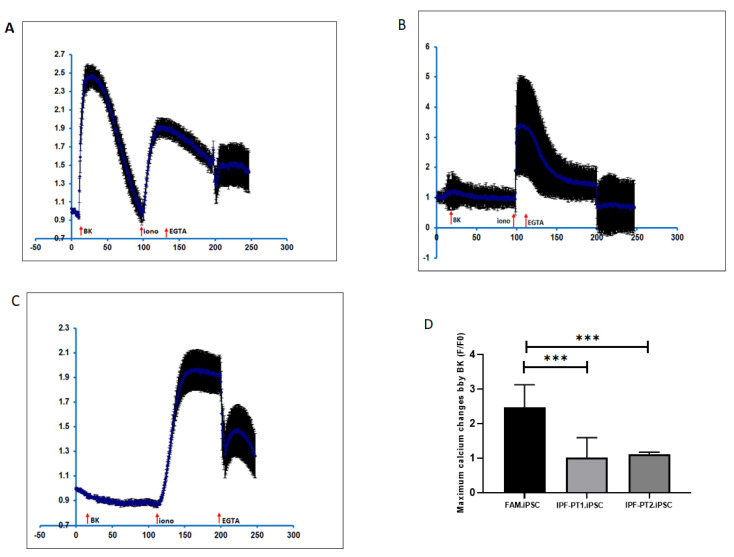
The effect of S100A3 and S100A13 mutations on intracellular calcium changes following the stimulation of cultured control FAM.iPSCs (**A**), IPF-PT1.iPSCs (**B**), IPF-PT1.iPSCs (**C**). The cells were stimulated with bradykinin (50 µM) followed by ionomycin (2 µM) and EGTA (1 mM). The histograms show the maximum response to bradykinin (**D**). The experiments were performed on live single cells using confocal laser scanning microscopy. The data are expressed as mean ± SEM (*n* = 6 and 5 for the control and patient cells, respectively). The data are expressed as the normalized fluorescence intensity ratio (F/F0) relative to the averaged three images obtained prior to the addition of the stimulus and are representative of three independent experiments. *p*-values are indicated (*** *p* < 0.001). FAM-iPSCs is a control cell line generated from control male donor skin fibroblast.

The mitochondrial staining with MitoTracker Red CMXRos in IPF-PT1 and IPFT-PT2 exhibited a more punctuated fluorescence pattern compared to the mitochondria in the control FAM (Figure 2A). This observation was confirmed by flow cytometry, and IPF-PT1 and IPFT-PT2 appeared to have more mitochondrial staining than FAM (Figure 2B). Based on our findings in [39,40], the S100A3 and S100A13 gene mutations affected intracellular calcium homeostasis. Therefore, when IPF-PT1 and IPFT-PT2 were stimulated with bradykinin (50 µM), intracellular calcium was significantly reduced compared to the control FAM-iPSC (Figure 3A–C).

### 3.3. Constitutive Secretion of IL-8, IL-6 and MCP-1 in Patient-Derived Fibroblasts

**Figure 4:**
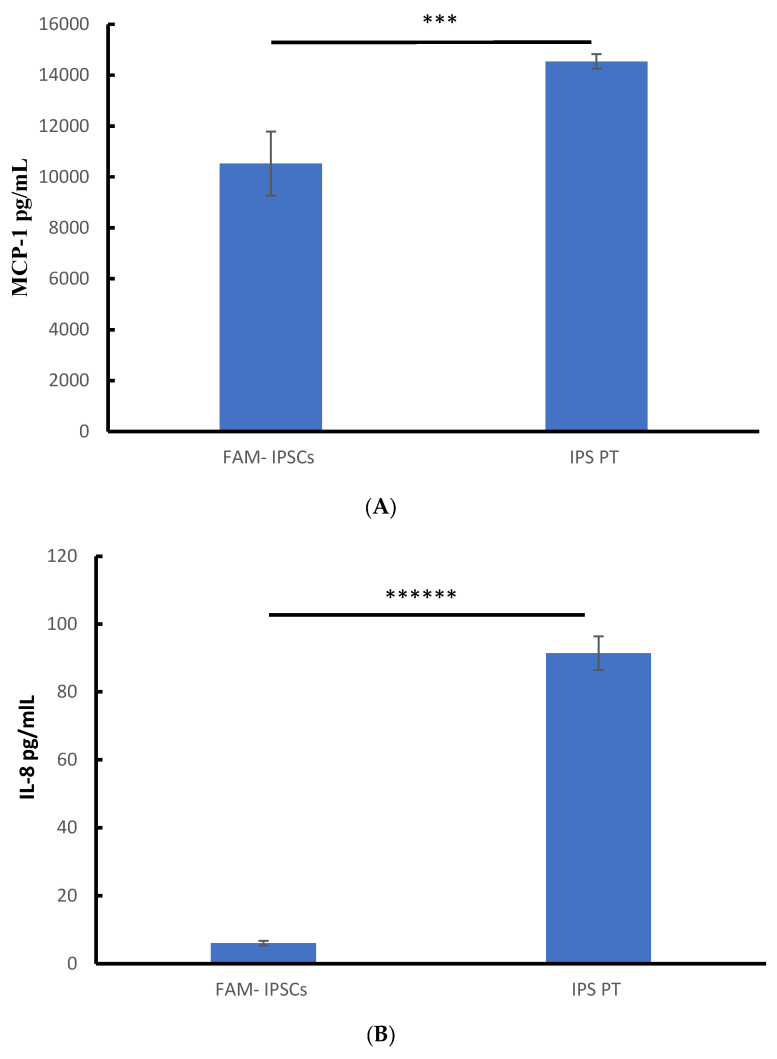

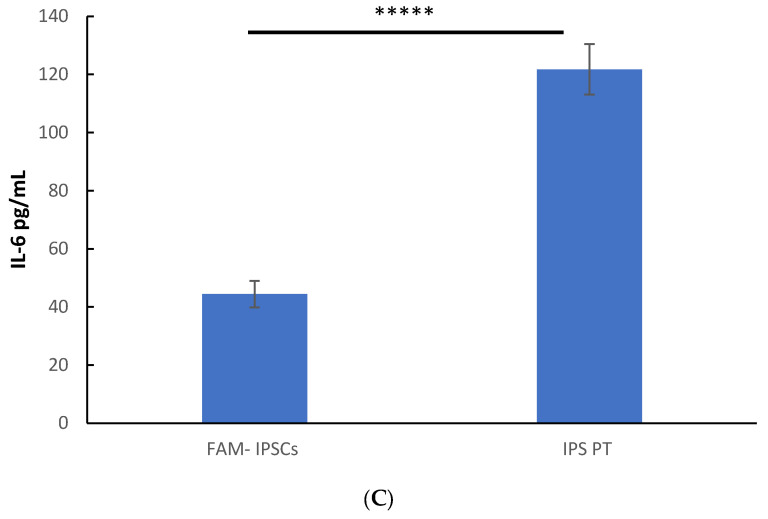
Inflammatory cytokine mediator secretion of patient-derived and normal donor iPSCs. (**A**–**C**) Constitutive release of MCP-1, IL-8, and IL-6 in the patient-derived iPSCs compared to the control-derived iPSCs (FAM). The experiments were performed on both patients (*n* = 3 each). *** *p* < 0.0001; ***** *p* < 0.000001; ****** *p* < 0.0000001.

The secretions of pro-inflammatory cytokines MCP-1, IL-8, and IL-6 were all substantially higher in the patient-derived cells (14,536.7 ± 286.47 pg/mL, 914,203 ± 4.9288 pg/mL, and 121.71997 ± 8.68 pg/mL) compared to the control samples (10527.2 ± 1259.76 pg/mL, 6.033266 ± 0.699 pg/mL, and 44.433798 ± 4.56 pg/mL) for MCP-1, IL-8, and IL-6, respectively. The experiments were conducted in triplicate on the control cells (FAM) and the patient cells. The patient cells represent the average pooled data from the IPF-PT1.iPSCs and IPF-PT2.iPSCs cell lines (*n* = 3 each) as demonstrated in (Figure 4). 

**Figure 5:**
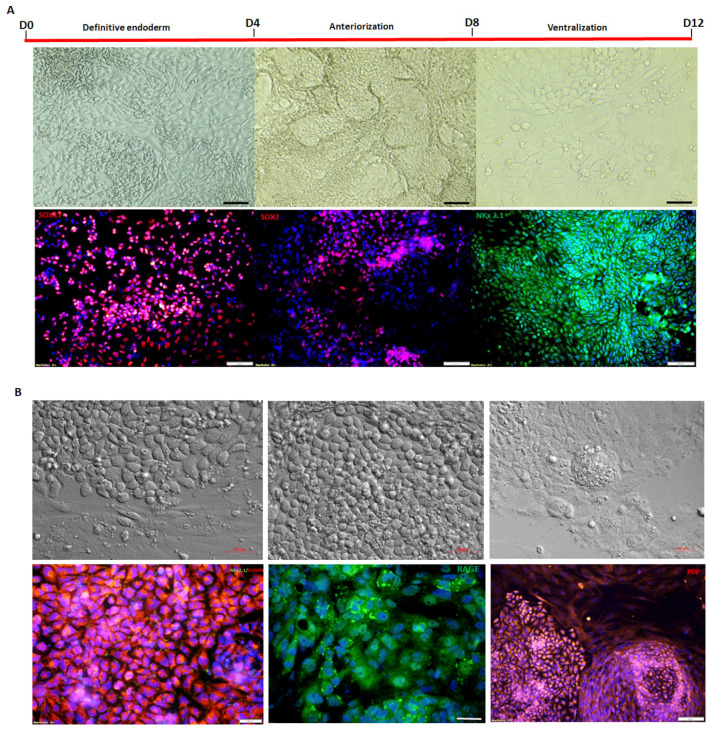

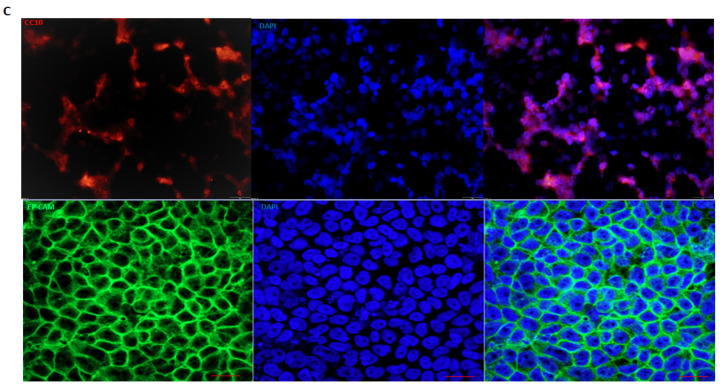
Putative lung epithelial cells: (**A**) Cells expressing definitive endoderm marker SOX17, followed by anteriorization (day 8) cells expressing SOX2, and finally by day 13 Nkx2.1 progenitor (Nkx 2.1 ^++^) cell expression. (**B**) Day 30, after the maturation of the progenitor cells into epithelial cells expressing markers such as podaplanin and RAGE, with the disappearance of Nkx2.1 ^++^ cells. (**C**) By day 50, the cells showed maturation toward lung epithelial cells by expressing EP-CAM membranous epithelial cell adhesion molecule and CC10, a non-ciliated airway lung epithelial cell protein. Scale bar: 10 µm.

**Figure 6:**
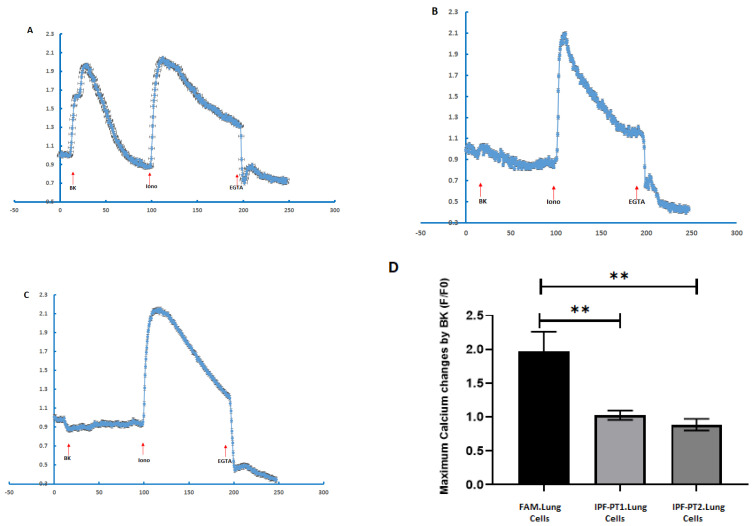
The effect of S100A3 and S100A13 mutations on intracellular calcium changes following stimulation of the cultured control FAM.iPSC lung cells (**A**), the IPF-PT1.iPSC lung cells (**B**), or the IPF-PT1.iPSC lung cells (**C**). The cells were stimulated with bradykinin (50 µM) followed by ionomycin (2 µM) and EGTA (1 mM). The histograms show the maximum response to bradykinin (**D**). The experiments were performed on live single cells using confocal laser scanning microscopy. The data are expressed as mean ± SEM (*n* = 4). The data are expressed as the normalized fluorescence intensity ratio (F/F0) relative to the averaged three images obtained prior to the addition of the stimulus and are representative of three independent experiments. ** *p* < 0.01.

The used protocol resulted in the sequential in vitro differentiation of the iPSC lines into foregut endoderm (SOX17 ^++^ cells expression), followed by anteriorization (SOX2 ^++^ cells expression), and finally, by day 13, Nkx2.1 progenitor (Nkx 2.1 ^++^ cells expression) (Figure 5A). After 30 days of differentiation, the cells began to express basal epithelial markers like podoplanin and RAGE (receptor for advanced glycation end products), a specific differentiation marker for human alveolar epithelial type I cells. This was accompanied by the disappearance of Nkx2.1^++^ cells, signifying the maturation of the progenitor cells into epithelial cells (see Figure 5B). By day 50, the cells showed maturation toward lung epithelial cells, expressing EP-CAM membranous epithelial cell adhesion molecule and CC10, a non-ciliated airway lung epithelial cell protein (Figure 5B,C). When measuring intracellular calcium in response to bradykinin (50 µM), the IPF-PT1- and IPF-PT2-generated epithelial lung cells were significantly reduced (Figure 6B,C) compared to the control FAM-generated epithelial lung cells (Figure 6A).

## 4. Discussion

The induced pluripotent stem cell platform has been under development for almost two decades, opening new frontiers for disease modeling and therapeutic development. Research on somatic cell reprogramming has identified the immense complexity of the cellular transformations occurring during iPSC induction and the role of transcriptional factors in the epigenetic regulation of cell fate. Therefore, the development of an iPSC-based cellular model for a range of diseases, from genetic to sporadic and age-related disorders, enables disease testing and prognosis, along with further therapeutic options. For this purpose, sophisticated cellular models, such as organs-on-a-chip and organoids, have been developed to study higher-order tissue architecture, compartmentalization, and long-range interactions in human development [55,56,57]. Consequently, we can use this in evaluating drug toxicity, efficacy, and pharmacokinetics, thus offering a preclinical general drug screening platform [58,59,60,61]. The first step in enhancing the translation of preclinical studies to the clinic is to develop screens based on hiPSC-derived cells—preferably of autonomous and disease-relevant phenotypes [62,63,64].

Here, we used the Sendai virus delivery system to generate induced pluripotent stem cells (iPSCs) from the peripheral skin fibroblast cells of a control and two patients with idiopathic pulmonary fibrosis (IPF). The generated iPSCs carried the mutation leading to diminished levels of calcium-binding protein S100A3 (NM_002960) and S100A13 (NM_001024210) expression. The cells showed altered calcium homeostasis, due to the mutation, and increased cristae damage of mitochondria compared to the control iPSCs. Moreover, the inflammatory mediators showed significantly higher levels of cytokines IL-8, IL-6, and MCP-1 in the patient-derived cells compared to the control-derived cells [49,65,66]. Although cytokine levels have been linked to pulmonary fibrosis [66], their source has typically been attributed to the activation network of pulmonary macrophages M1/M2 [67]. Even in the absence of macrophages or any other cell type, the patient’s fibroblasts alone can constitutively produce inflammatory cytokines, directly implicating S100A3/S100A13 mutations in the observed cytokine secretion [12]. In a further step, the cells were successfully differentiated into lung epithelial cells to provide a platform for studying the disease [68,69,70,71,72]. The lung epithelial cells generated from the patients’ iPSCs exhibited reduced calcium homeostasis compared to the cells generated from the control iPSCs. The mutation in S100A13 is a frameshift mutation that is expected to produce a truncated form of the protein [39], which explains its pathogenicity. In this scenario, treatment with recombinant S100A13 protein or transfection with wild-type S100A13 alone normalized the calcium levels in the patient-derived cells to those comparable to the control cells [13]. The interaction between these two calcium-binding proteins in contributing to the fibrosis phenotype remains to be elucidated. The performance of calcium-based therapy studies using the generated iPSCs as a disease model is a major goal, as this would help to speed up clinical application. In a drug discovery setting, having a disease model or translational platform is essential for the research team to predict candidate molecules’ efficacy and toxicity. It is not necessary that every aspect of human disease pathology be recapitulated, but a chain of translatability can be established from preclinical models to the human population for the intended treatment [73,74,75]. This iPSC disease model is the first to replicate the role of S100A3 and S100A13 in lung fibrosis. Utilizing patient-derived cells will aid in drug development, enhancing the translation of preclinical studies to clinical applications. The demand for commercial organoids will likely increase in the next few years. In 2019, six companies were active, and nineteen others expressed an interest in cancer, renal, gut, lung, and neural organoids.

## 5. Concluding Remarks

In conclusion, the generated IPSCs–alveolar progenitors recapitulate the disease model and present a platform for further disease prognosis studies and personalized therapy testing. The next step involves refining the maturation and differentiation of epithelial cells within the organoid model. This model will be used to further explore the interaction between the proteins S100A3 and S100A13 in the context of fibrosis. The preliminary results suggest that treating patients’ cells with recombinant S100A3 and S100A13 proteins effectively normalizes most cellular responses. Consequently, these findings may support the potential use of these recombinant proteins in treating this severe disease.

The current advancements in iPSC organoid technologies highlight the significance of these platforms in identifying new cellular and molecular modulators that are crucial for the development, progression, and resolution of respiratory diseases. 

## Data Availability

No new data were created or analyzed in this study.

## Supplementary Materials

The following supporting information can be downloaded at: https://www.mdpi.com/article/10.3390/cells14110781/s1, Supplementary S1: Molecular analyses in pulmonary fibrosis of three iPSC lines; Normal wild Type (FAM), IPF-PT1, and IPF-PT2; Supplementary S2: Effect of S100A3 and S100A13 mutations on protein expression.
