# Supplementary material for: The Generation of Two Induced Pluripotent Cell Lines from Patients with an Atypical Familial Form of Lung Fibrosis
Source: Cells. 2025 May 26;14(11):781. doi: 10.3390/cells14110781 (PMC12153737; doi:10.3390/cells14110781)
Supplement: Supplementary file 1 [file cells-14-00781-s001.zip › cells-3629954-supplementary.pdf]

Supplementary materials

A)

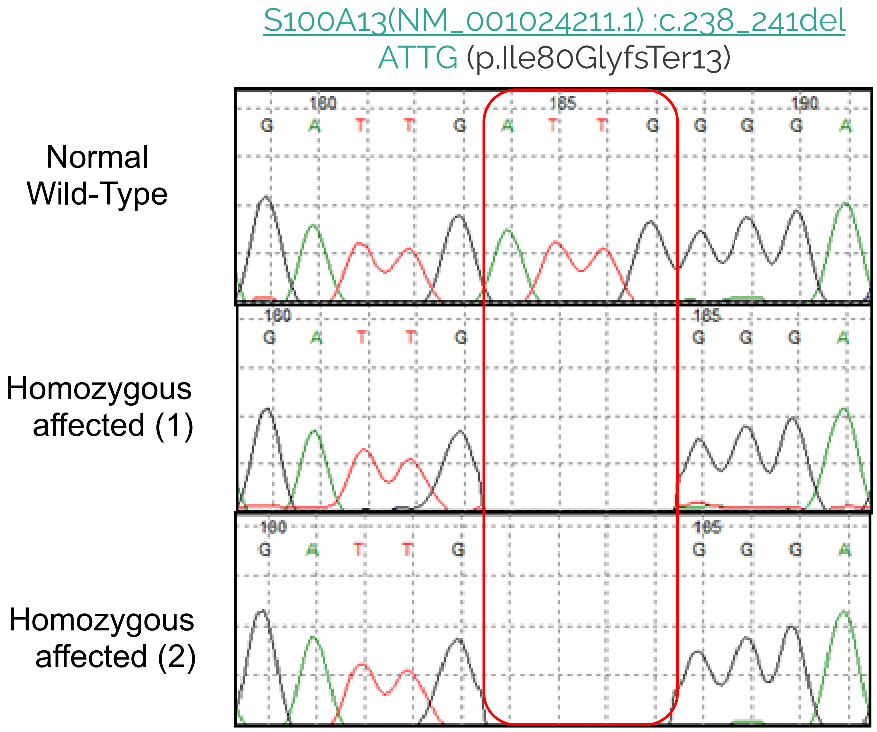

B)

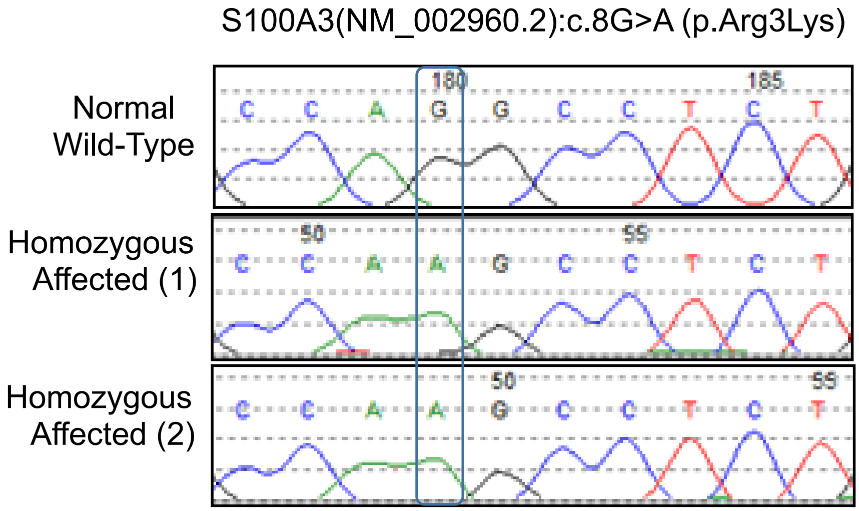

Supplementary S1: Molecular analyses in pulmonary fibrosis of three iPSC lines; Normal wild Type (FAM), IPF-PT1, and IPF-PT2. A, B) Sequence chromatograms indicating the wild-type (FAM), and homozygous affected forms of IPF-PT1, and IPF-PT2**;** A) the c.238–241delATTG (p.Ile80GlyfsTer13) in S100A13.B) Transition at position c.8 changing the arginine residue to Lysine at position 3 of the S100A3 protein (c.8G>A (p.Arg3Lysc)) comparing to wild type (FAM) cells. Mutation name is based on the full-length S100A3 (NM_002960.2) and S100A13 (NM_001024211.1) transcripts.

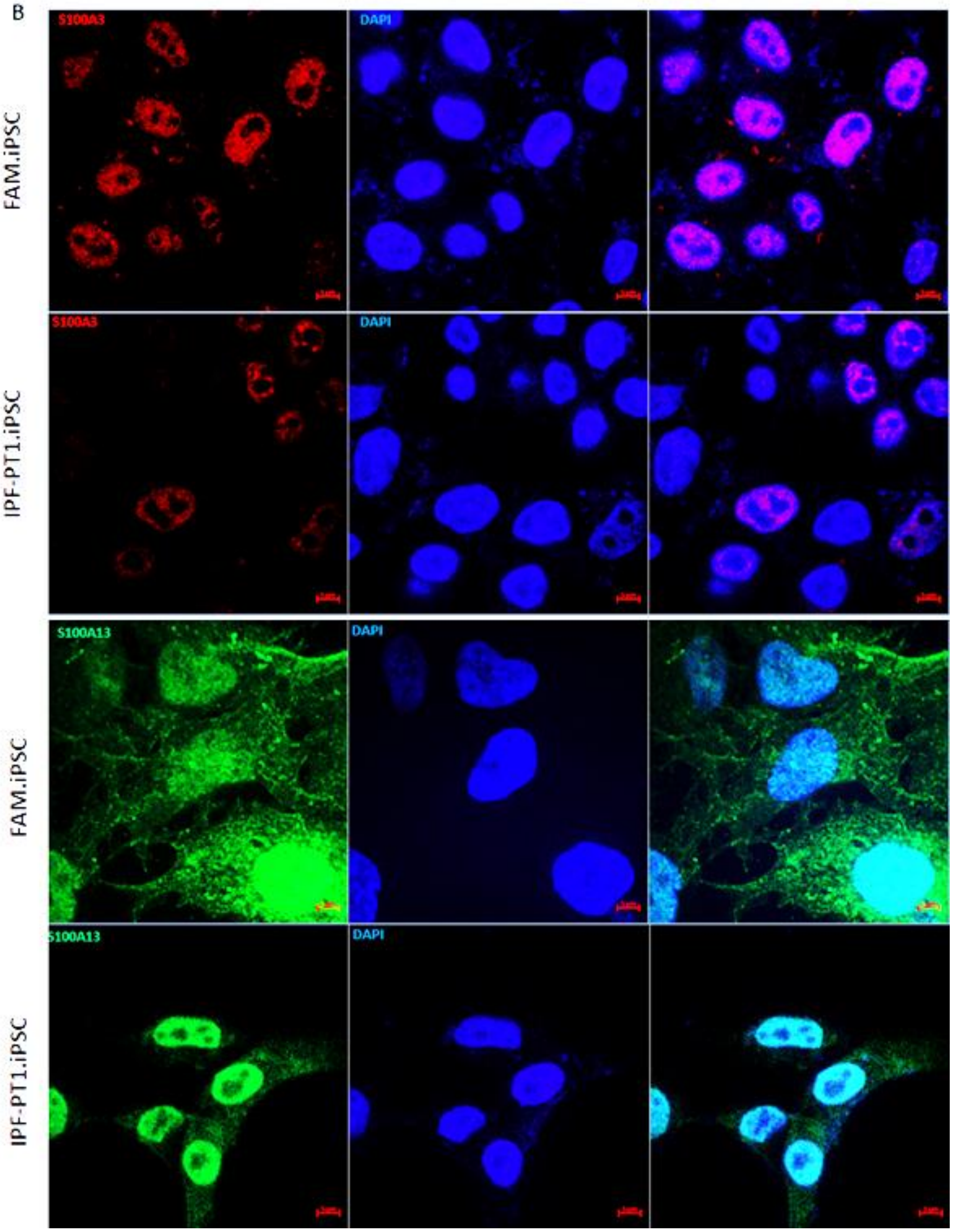
B
S100A3
DAPI
FAM.iPSC
S100A3
DAPI
IPF-PT1.iPSC
S100A13
DAPI
FAM.iPSC
S100A13
DAPI
IPF-PT1.iPSC

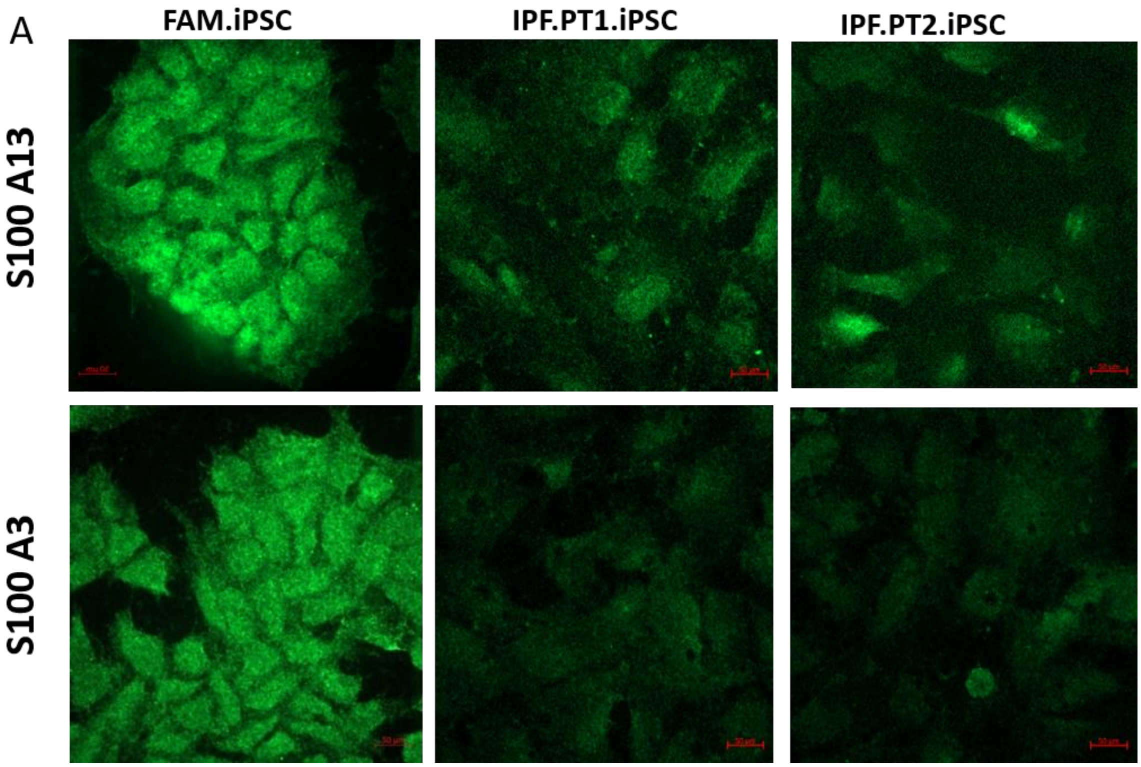

Supplementary S2 Effect of S100A3 and S100A13 mutations on protein expression. (A)&(B) Confocal fluorescence laser scanning micrographs showing the reduced expression of S100A3 and S100A13 proteins in iPSCs lines from patients compared with controls. Data are representative of three independent experiments with cells isolated from two patients and two controls. Scale bar: 50 μm.
